# Supplementary material for: Earthquake crisis unveils the growth of an incipient continental fault system
Source: Nat Commun. 2019 Sep 2;10:3482. doi: 10.1038/s41467-019-11064-5 (PMC6718684; doi:10.1038/s41467-019-11064-5)
Supplement: Supplementary file 1 — Supplementary Information [file 41467_2019_11064_MOESM1_ESM.pdf]

Earthquake crisis unveils the growth of an incipient continental fault system

Gràcia et al.

Supplementary Figures

This document contains supplementary figures and tables referred to in the main text and Methods of the article.

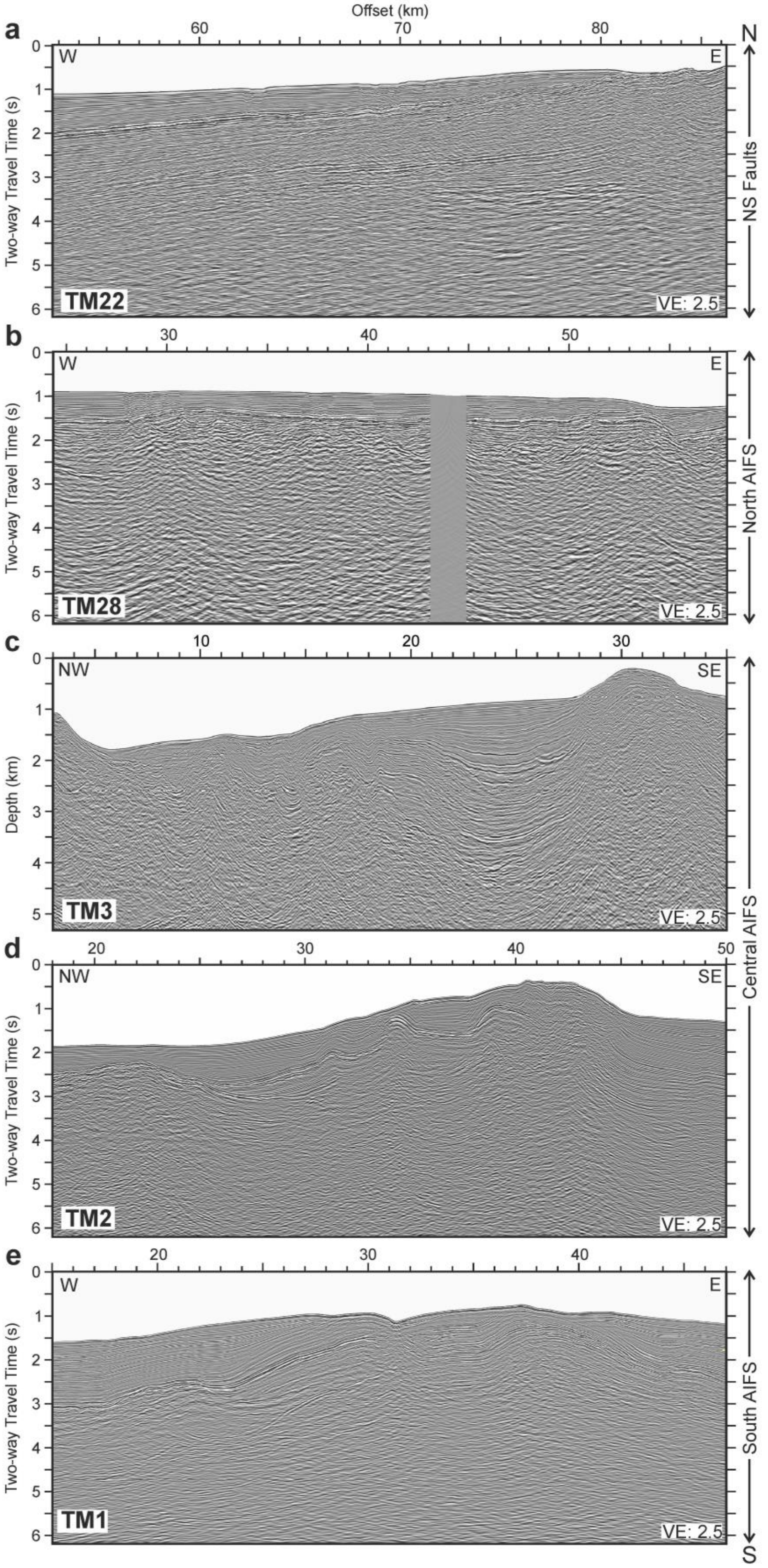

Supplementary Figure 1. **High-resolution copies of the non-interpreted seismic reflection profiles.**

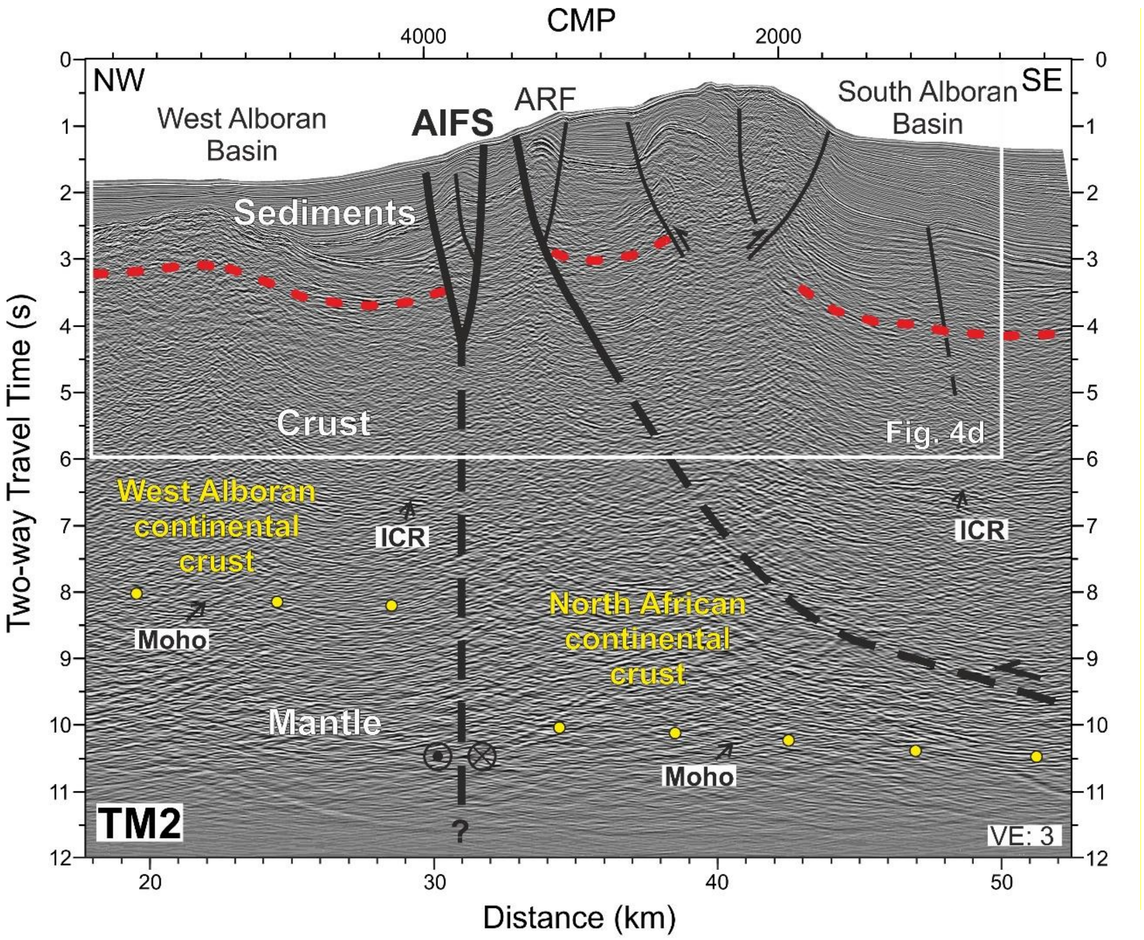

Supplementary Figure 2. **Multichannel seismic (MCS) profile TM2 demonstrating that the AIFS is a crustal scale structure**. Profile TM2 reaches down to 12s (TWTT) and the main layers (i.e. sediments, crust and mantle) are defined. Red dashes mark the boundary between stratified sediments and upper crust, while yellow dots depict the Moho boundary between the lower crust and upper mantle. Main tectonic structures, Alboran Ridge Fault and AIFS (Al-Idrissi Fault System) are also depicted. Differences in crustal thickness across AIFS (i.e. 8 s TWTT in West Alboran crust and 10.5 s TWTT in North African crust) are identified. ICR: Intra-crustal reflections. White rectangle depicts profile TM2 as shown in Figure 4d. This is supported by a recent work with local earthquake tomography and with a better ray coverage of the offshore region.

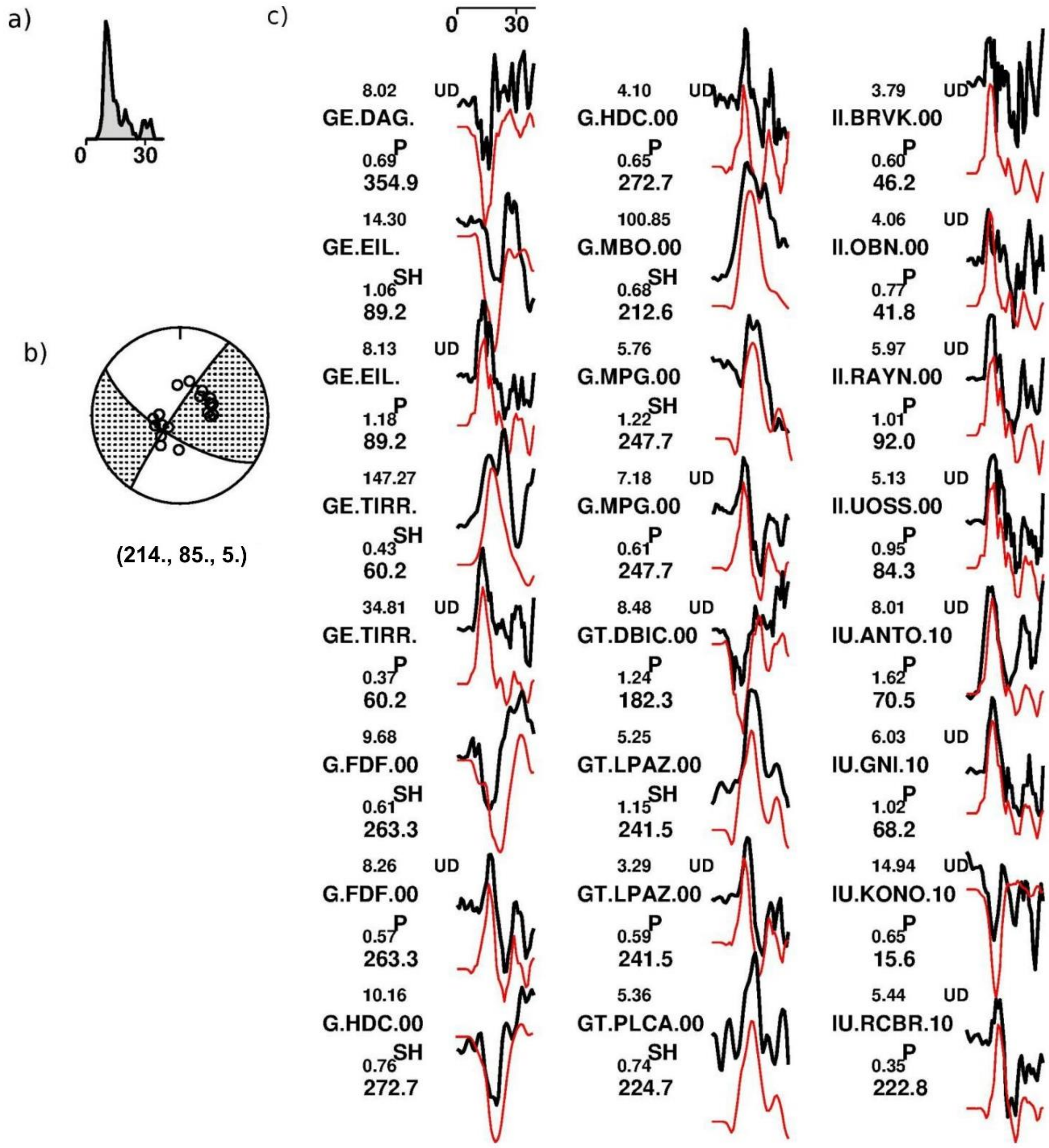

Supplementary Figure 3. **Source time function, focal mechanism and P and SH waveform inversion of the 25 January 2016 earthquake**. a) Moment tensor inversion indicates a minimum misfit at 10 km depth. b) Nodal plane corresponds to a left-lateral strike-slip focal mechanism with a preferred nodal plane of 214º/85º/5º (strike/dip/rake). c) P and SH waveform fits for the slip inversion of the 2016 earthquake. Data for each station is shown by a thick black line and synthetics by a thin red line.

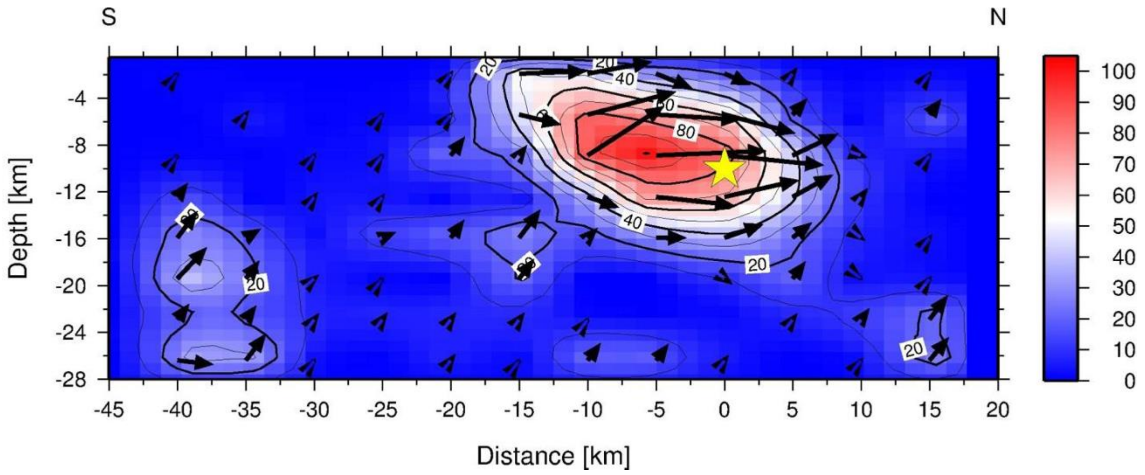

Supplementary Figure 4. **Coseismic slip (in centimeters) determined from the inversion of the teleseismic waveforms,** yielding shallow slip and hence potential surface rupture in the vicinity of the epicenter of the 25 January 2016 earthquake. The yellow star indicates the centroid of the point source approximation of the mainshock. Slip direction is indicated by arrows, favoring a northward propagation of the rupture front. Note that the lengths of arrows correspond to slip magnitude. Please, note that hypocentre location of the mainshock (yellow star) and centroid depth or maximum of slip are shifted with respect to each other by ~8 km. The offset is controlled by differences in location procedures. Thus, all earthquake hypocentres are derived from regional observations at the IGN network, using a local velocity model and station corrections for the Alboran domain (see Methods). In contrast, slip inversion uses observations at large epicentral distances (30-90°) and a global 1-D velocity model (see Methods)

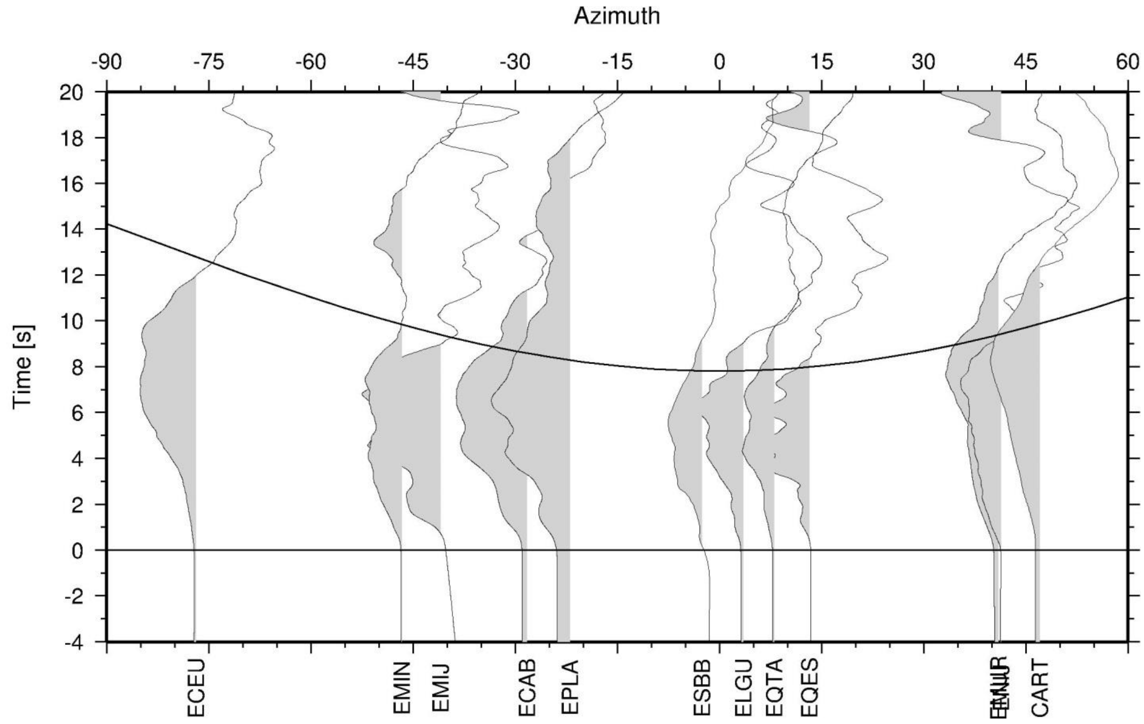

Supplementary Figure 5. **Directivity effect of the 25 January 2016 mainshock from regional seismic waveforms**. Depending on the location of a seismic station, the apparent source duration of the onset depends on the direction of rupture. Shorter times mark stations located in the rupture direction and hence support northward rupture propagation. The solid line indicates apparent rupture time $t(\phi)$ of a roughly southward $\phi=0$ propagating slip and is related to $t(\phi)= t_r + L/vr – L/vp \cos(\phi)$, where t_r is the rise time, L is the length of the fault plane, v_r is the rupture velocity, v_p is the P-wave velocity, and ϕ is the azimuth. Station codes are given below each waveform.

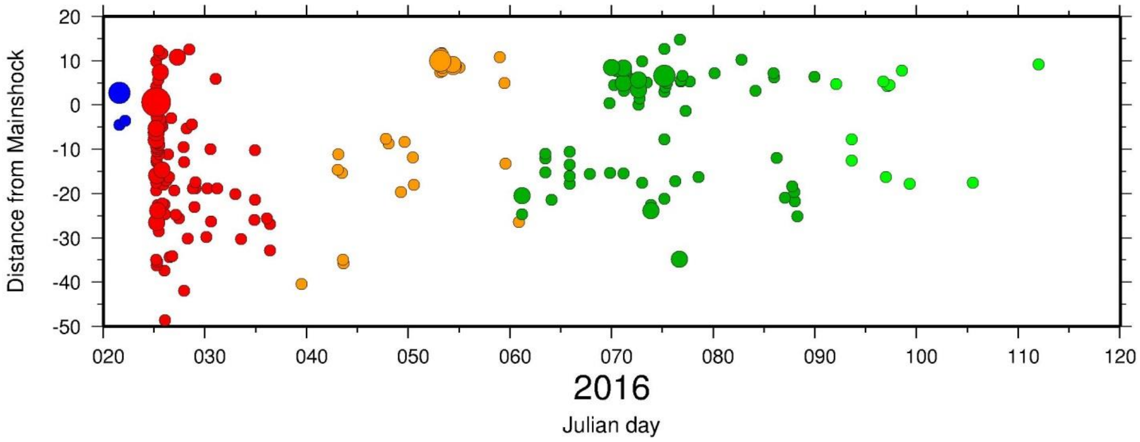

Supplementary Figure 6. **Distribution of seismicity as a function of Julian day and distance from the epicenter of the mainshock along a NE-SW trending profile**. Blue: pre-shock activity; red mainshock (large red dot) and earlier aftershocks; orange to light green later aftershocks. Note that the mainshock area shows little aftershock activity, while activity was greatest to the north and south. Furthermore, activity seems to migrate to the NE after day 50.

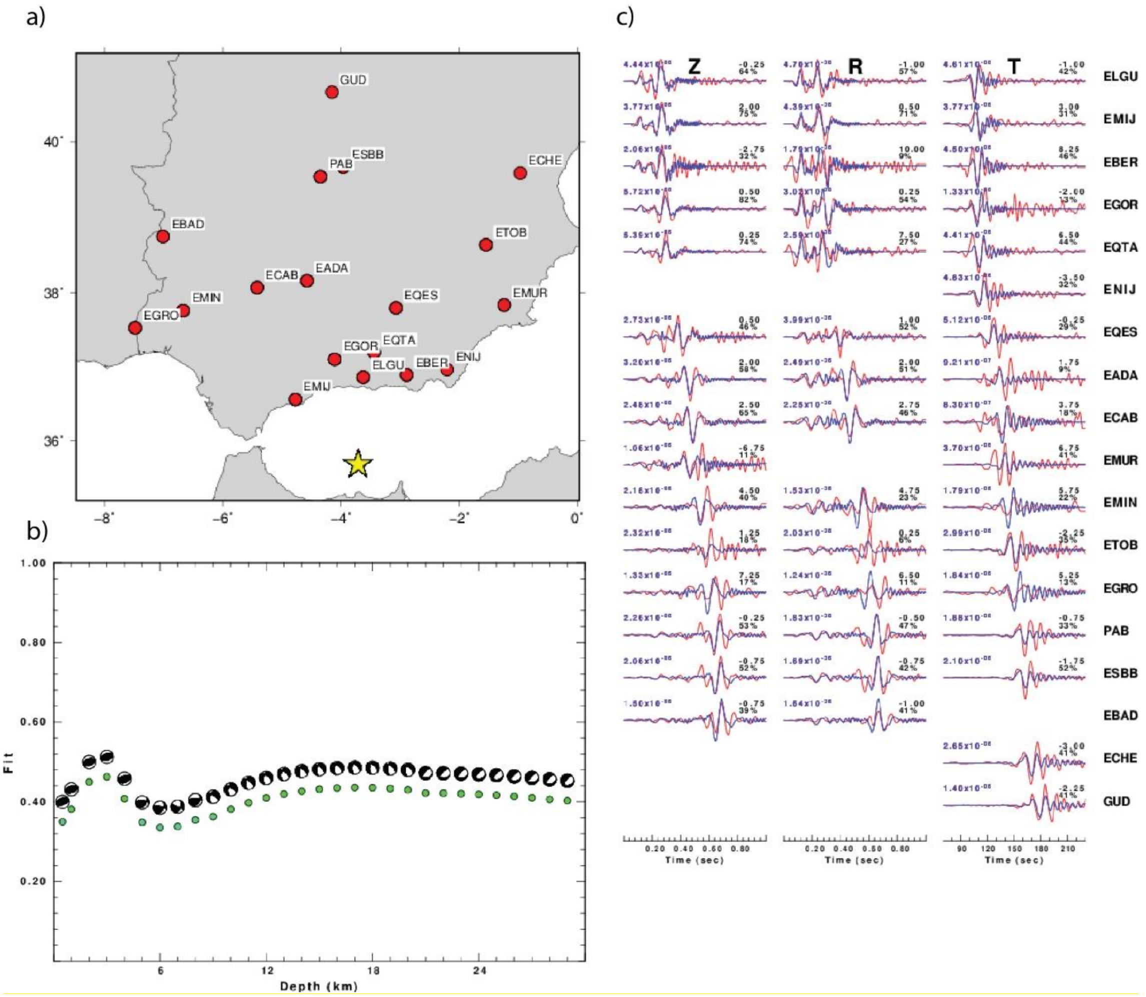

Supplementary Figure 7. **Example of a regional moment tensor solution for the aftershock at 2016-01-25 14:52:41 UTC with M_W 4.5 (8th event in Supplementary Table 1)**. a) Map with the location of the earthquake (yellow star) and stations used to obtain the solution (labelled red circles). b) Results of the grid search for different values of focal depth. The solution with the highest value of the fit is the preferred focal depth (3 km in this case). Each beach ball indicates the best double-couple focal mechanism for each depth. c) Comparison between the observed seismograms (red) and synthetic seismograms (blue) corresponding to the best moment tensor solution. Z indicates the vertical component, R the radial component, and T the transverse component. The data repository can be download at:

https://digital.csic.es/handle/10261/177887
http://dx.doi.org/10.20350/digitalCSIC/8623

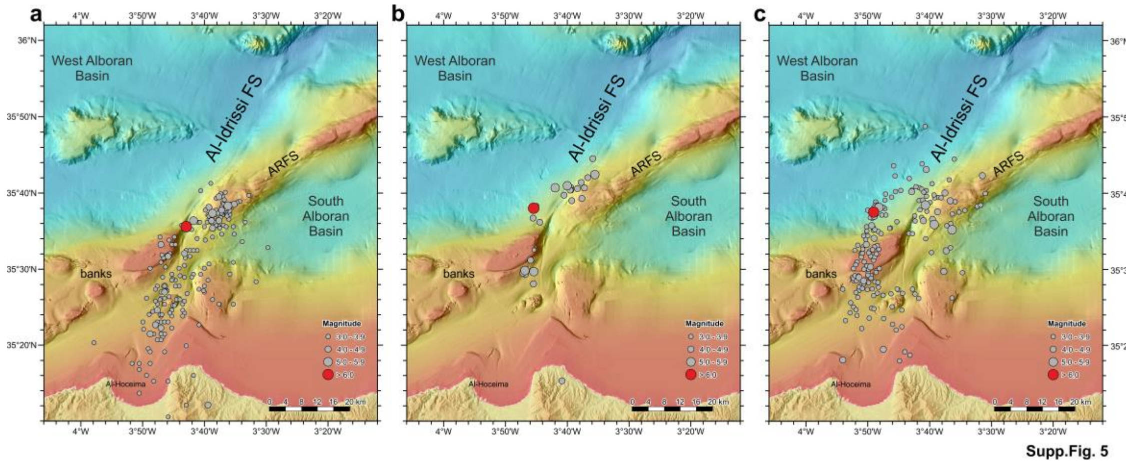

Supplementary Figure 8. **Comparison of the mainshock and aftershock locations based on different velocity models.** a) In the left image, our results show how the mainshock (in red) and aftershocks (in grey) are relocated using a local lithospheric velocity model based on onshore-offshore stations. The mainshock and aftershocks fall on the trace of the Al-Idrissi Fault System. b) On the central image, the main shock (in red) and aftershocks (in grey) fall relatively close (~4 km) to the trace of the Al-Idrissi Fault System, obtained with a regional 3D velocity model (Buforn et al, 2018)[13]. c) On the right, the mainshock (in red) and aftershocks (in grey) are relocated using a 1D velocity model for Iberia (IGN catalogue), which locates the mainshock and most of the aftershocks 15 km west of the fault trace[15,39].

Supplementary Tables

Supplementary Table 1. **Moment tensor solutions of the largest events[a] that occurred in January-February 2016**.

Date (day/month/year)	Origin time (h:min)	Latitude (ºN)	Longitude (ºE)	Depth (km)	Magnitude Mw	Strike (º)	Dip (º)	Rake (º)
21/01/2016	13:47	35.60	-3.70	15	5.0	300	80	25
25/01/2016	05:22	35.59	-3.72	10	6.4	214	85	5
25/01/2016	05:54	35.45	-3.77	17	5.0	180	80	-15
25/01/2016	06:10	35.36	-3.81	16	5.1	160	75	-30
25/01/2016	06:30	35.36	-3.77	15	4.2	130	65	30
25/01/2016	07:55	35.36	-3.80	16	4.1	100	40	-10
25/01/2016	08:25	35.37	-3.78	14	4.2	125	65	30
25/01/2016	14:52	35.63	-3.65	3	4.5	250	45	90
25/01/2016	16:02	35.45	-3.80	10	4.1	160	75	50
25/01/2016	18:17	35.46	-3.77	17	4.1	285	90	10
26/01/2016	01:16	35.33	-3.71	15	4.1	200	45	-35
26/01/2016	04:35	35.42	-3.74	25	4.4	360	15	80
26/01/2016	23:15	35.44	-3.82	16	4.2	115	55	20
27/01/2016	06:32	35.66	-3.63	12	4.4	295	50	45
27/01/2016	21:57	35.49	-3.70	10	4.2	175	65	-40
27/01/2016	22:10	35.46	-3.73	10	4.2	330	80	20
28/01/2016	19:48	35.40	-3.72	10	4.2	175	60	80
05/02/2016	09:02	35.30	-3.81	14	3.6	140	55	75
22/02/2016	03:46	35.64	-3.60	3	4.9	245	40	85
22/02/2016	04:14	35.64	-3.59	3	4.0	240	40	85
23/02/2016	08:46	35.63	-3.60	3	4.1	65	45	90
23/02/2016	10:12	35.63	-3.61	16	4.2	185	65	-30

[a] The strike, dip and rake of the preferred nodal planes are given.
