# Supplementary material for: Earthquake crisis unveils the growth of an incipient continental fault system
Source: Nat Commun. 2019 Sep 2;10:3482. doi: 10.1038/s41467-019-11064-5 (PMC6718684; doi:10.1038/s41467-019-11064-5)
Supplement: Supplementary file 4 — Source Data [file 41467_2019_11064_MOESM4_ESM.zip › repositorydata.docx]

**TOPOGRAPHY:**

NASA JPL (2013).*NASA Shuttle Radar Topography Mission Global 1 arc second* [Data set]. NASA EOSDIS Land Processes DAAC.

doi: 10.5067/MEaSUREs/SRTM/SRTMGL1.003

**BATHYMETRY:**

EMODnet Bathymetry Consortium (2018): EMODnet Digital Bathymetry (DTM).

<http://doi.org/10.12770/18ff0d48-b203-4a65-94a9-5fd8b0ec35f6>

**We include two files:**

F4_2018.xyz

F4_2018_rgb.tif

(see EMODNet-bathymetry.zip file)

*****************************************************

**MULTICHANNEL-SEISMIC REFLECTION PROFILES:**

**We include three files:**

TM02-cortical.pdf

TM22-cortical.pdf

TM28-cortical.pdf

(see SEISMICS.zip file)

*****************************************************

**Regional centroid moment tensors**

The repository of regional centroid moment tensors can be downloaded at this link:

<https://digital.csic.es/handle/10261/177887>

http://dx.doi.org/10.20350/digitalCSIC/8623

*****************************************************

**data from IRIS data centrE**

This work included data from the II, IU, GE, GT, and G seismic networks obtained from the IRIS data centre

<http://dx.doi.org/doi:10.7914/SN/II>; <http://dx.doi.org/doi:10.7914/SN/IU>; <http://dx.doi.org/doi:10.14470/TR560404>; <http://dx.doi.org/doi:10.7914/SN/GT>; <http://dx.doi.org/doi:10.18715/GEOSCOPE.G>

**DATA FROM THE IGN CATALOGUE**

<http://www.ign.es/web/en/ign/portal/sis-catalogo-terremotos> (last visited January 2018).
