# Supplementary figures and images for: Earthquake crisis unveils the growth of an incipient continental fault system
Source: Nat Commun. 2019 Sep 2;10:3482. doi: 10.1038/s41467-019-11064-5 (PMC6718684; doi:10.1038/s41467-019-11064-5)

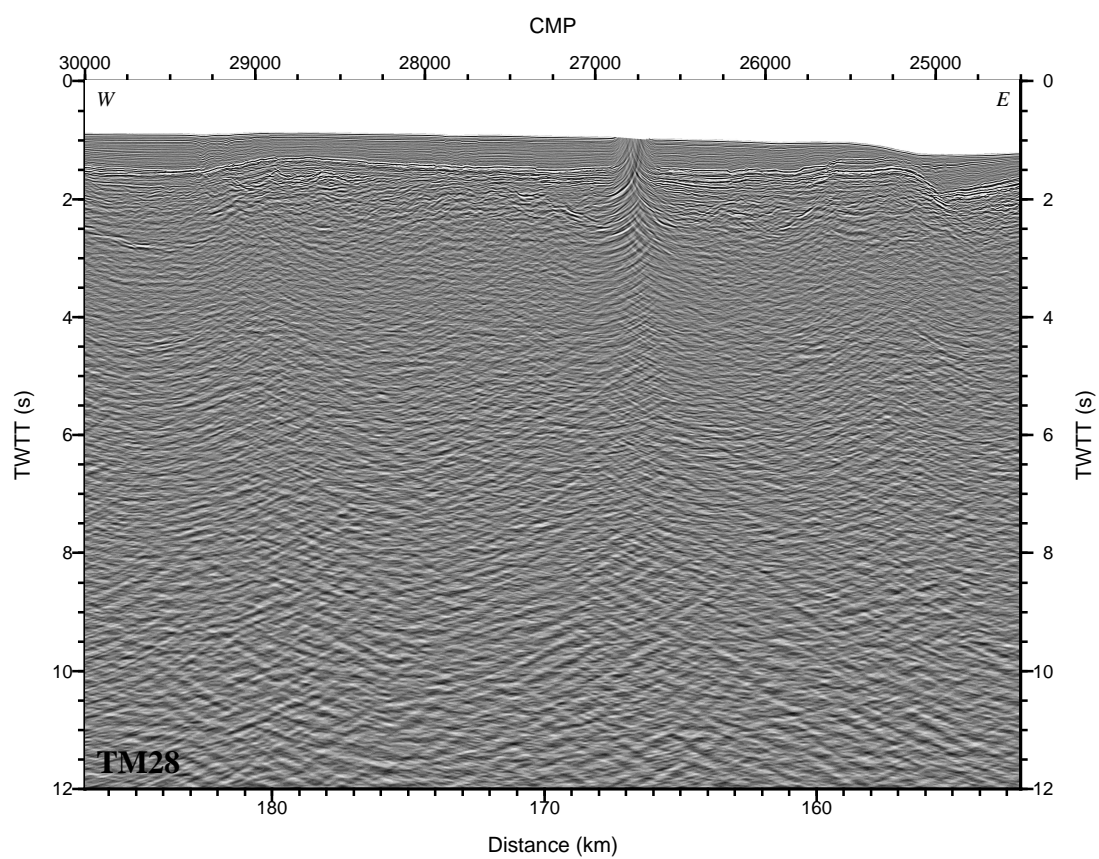

Supplement: Supplementary file 4 — Source Data [file 41467_2019_11064_MOESM4_ESM.zip › 1.MULTICHANNEL-SEISMIC REFLECTION PROFILES.pdf]

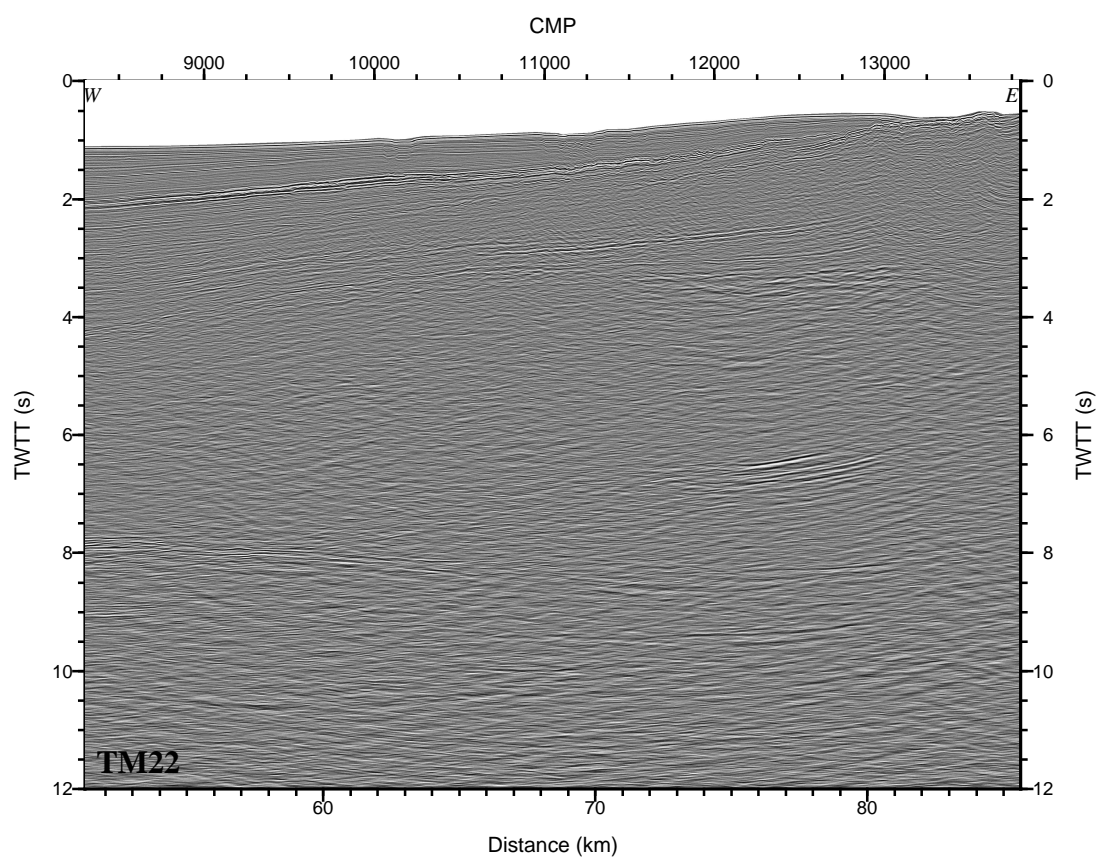

Supplement: Supplementary file 4 — Source Data [file 41467_2019_11064_MOESM4_ESM.zip › 2.MULTICHANNEL-SEISMIC REFLECTION PROFILES.pdf]

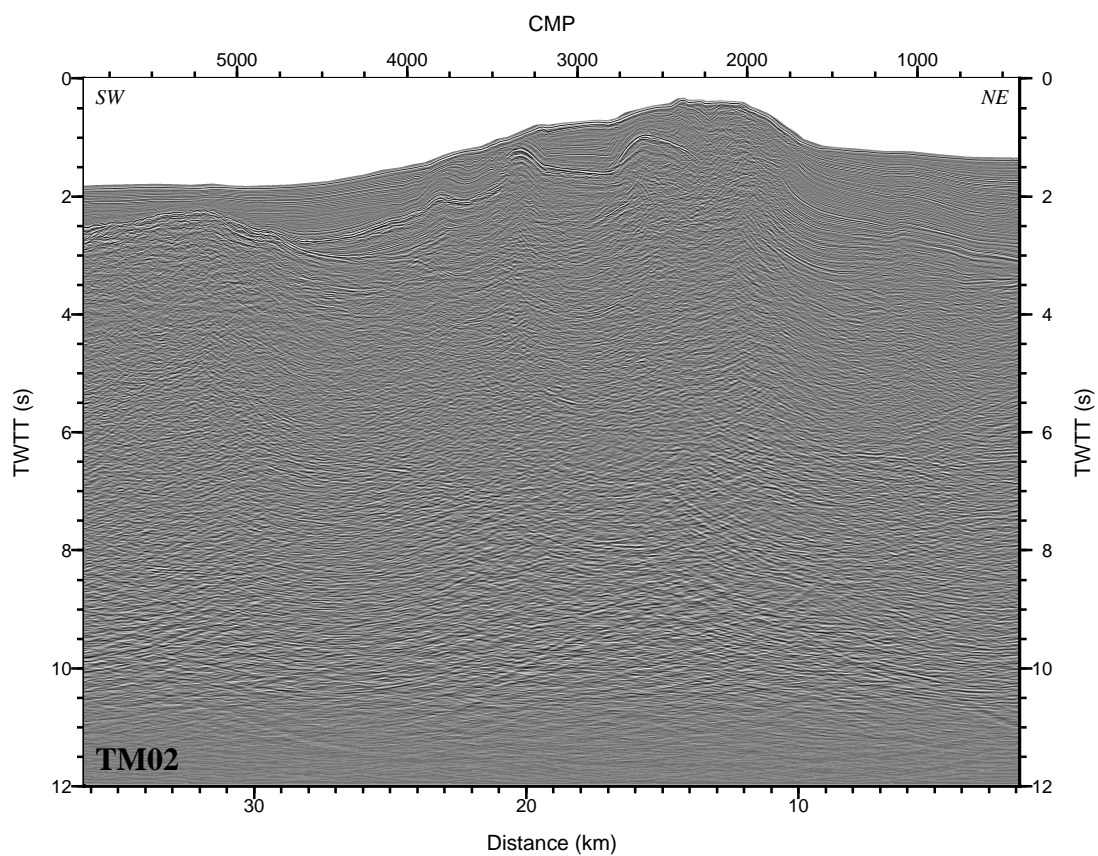

Supplement: Supplementary file 4 — Source Data [file 41467_2019_11064_MOESM4_ESM.zip › 3.MULTICHANNEL-SEISMIC REFLECTION PROFILES.pdf]
